# N-acetylcysteine does not prevent contrast nephropathy in patients with renal impairment undergoing emergency CT: a randomized study

**DOI:** 10.1186/1471-2369-14-119

**Published:** 2013-06-03

**Authors:** Pierre-Alexandre Poletti, Alexandra Platon, Sophie De Seigneux, Elise Dupuis-Lozeron, François Sarasin, Christoph D Becker, Thomas Perneger, Patrick Saudan, Pierre-Yves Martin

**Affiliations:** 1 Department of Radiology, University Hospital of Geneva, Geneva, Switzerland; 2 Service of Nephrology, Department of Internal Medicine specialties, University Hospital of Geneva, Geneva, Switzerland; 3 Division of Clinical Epidemiology, University Hospital of Geneva, Geneva, Switzerland; 4 Service of Emergency Medicine, University Hospital of Geneva, Geneva, Switzerland

**Keywords:** Computerized Tomography, Contrast media, Emergency medicine, N-acetylcysteine, Nephrotoxicity

## Abstract

**Background:**

Patients admitted to the emergency room with renal impairment and undergoing a contrast computed tomography (CT) are at high risk of developing contrast nephropathy as emergency precludes sufficient hydration prior to contrast use. The value of an ultra-high dose of intravenous N-acetylcysteine in this setting is unknown.

**Methods:**

From 2008 to 2010, we randomized 120 consecutive patients admitted to the emergency room with an estimated clearance lower than 60 ml/min/1.73 m^2^ by MDRD (mean GFR 42 ml/min/1.73 m^2^) to either placebo or 6000 mg N-acetylcysteine iv one hour before contrast CT in addition to iv saline. Serum cystatin C and creatinine were measured one hour prior to and at day 2, 4 and 10 after contrast injection. Nephrotoxicity was defined either as 25% or 44 μmol/l increase in serum creatinine or cystatin C levels compared to baseline values.

**Results:**

Contrast nephrotoxicity occurred in 22% of patients who received placebo (13/58) and 27% of patients who received N-acetylcysteine (14/52, p = 0.66). Ultra-high dose intravenous N-acetylcysteine did not alter creatinine or cystatin C levels. No secondary effects were noted within the 2 groups during follow-up.

**Conclusions:**

An ultra-high dose of intravenous N-acetylcysteine is ineffective at preventing nephrotoxicity in patients with renal impairment undergoing emergency contrast CT.

**Trial registration:**

The study was registered as Clinical trial (NCT01467154).

## Background

Prevention of acute kidney injury (AKI) post contrast injection remains mandatory given its association to morbidity and mortality [1].

Contrast nephropathy (CN) is classically defined as an increase in 25% or more, or as an absolute increase of 44 μmol/l of creatinine levels within three days following contrast injection [2]. Recently, AKIN (acute kidney injury network) and RIFLE (risk, injury, failure loss and end-stage kidney disease) criteria have emerged to standardize the diagnosis of AKI in hospitalized patients [3]. These criteria are important as they correlate to mortality in critically and non critically ill patients [4-6]. AKIN criteria have rarely been used for CN but appear to also predict mortality in critically ill patients [7]. Finally, other markers such as cystatin C or NGAL may be more sensitive to identify CN and also to predict mortality [8] although a definition of CN using these markers is currently lacking.

In patients undergoing elective radiological or cardiologic procedures, CN can be prevented by hydration, withdrawal of diuretics and/or nephrotoxic drugs. Its prevalence has therefore declined over the recent years [9]. Occurrence of CN is however consistently higher in diabetics, congestive heart failure patients, volume depleted patients, critically ill patients and especially in patients suffering from chronic kidney disease (CKD) [7,10-12]. Emergency CT with contrast injection in patients with renal impairment (eGFR < 60 ml/min) remains an important cause of contrast nephropathy given the lack of sufficient time for hydration and the lack of renal functional reserve. Finding an efficient prevention for contrast nephropathy in this specific population would be highly desirable.

N-acetylcysteine (NAC) may protect against contrast nephropathy [13]. While hydration is clearly beneficial in preventing CN [14], the role of NAC administration is still uncertain and results of randomized control trials gave conflicting results regarding its effect [15]. In addition, NAC does not seem to prevent AKI in patients with normal renal function [16]. Intravenous NAC may be the form of choice in emergency procedures given its rapid availability and its ease of administration in patients whose consciousness is altered or who cannot eat. However, when using the iv route in emergency, higher doses of NAC (6000 mg) may be necessary to obtain a preventive effect as tested by Marenzi and collegues [17]. Furthermore, the Rappid study also demonstrated that high dose iv NAC was efficient in prevention of CN, with doses derived from the one used in paracetamol intoxication [18]. Indeed, NAC administered by the intravenous route may be less effective in generating gluthatione than when given orally [19]. Finally, in a previous study performed in patients with elevated creatinine levels undergoing emergency CT-Scan, 1800 mg of NAC administered iv failed to prevent contrast induced nephropathy [20].

The aim of the present study was therefore to determine whether 6000 mg (ultra high dose) intravenous NAC, to account for a dose dependent effect, was efficient in preventing CN after emergency contrast CT-scan in patients admitted to the ER with elevated creatinine levels.

## Methods

From 2008 to 2010, 124 consecutive patients were eligible in the emergency room on account of an estimated creatinine clearance by MDRD [21] of less than 60 ml/min/1.73 m^2^ and a request for an urgent contrast CT. Exclusion criteria were asthma, pregnancy, obstructive nephropathy and patients’s refusal. Written consent was obtained from every patient and from a physician independent from the study. 4 patients refused to participate to the study. 120 patients were randomized to placebo or high dose iv NAC. The study was approved by the ethical committee of the University Hospital of Geneva (IRB 07–121) as well as by the Swiss Agency for Therapeutic Products (Swissmedic, N°2008DR4057).

Iohexol (Accupaque®, GE Healthcare, Opfikon) was the contrast medium used in this study at a dose of 1.5 ml/kg to a maximum of 150 ml.

### Study protocol

Patients were randomized to either placebo (0.45% saline) or high dose (6000 mg) iv NAC (Fluimicil®, Zambon, Cadempino, Switzerland) diluted in 100 ml 0.45% saline to be administered during the 60 minutes before the CT-Scan. The randomization list was generated by computer, and NAC or placebo vials were prepared and numbered accordingly by the pharmacy. Investigators, patients and patient’s primary physician were blinded to treatment group. No patients in the control group received NAC outside the study protocol.

Randomized patients were then assigned to hydration of 250 ml of 0.45% NaCl before the CT-Scan and blindly allocated to either high dose NAC versus placebo. After the CT-Scan, all patients received 1000 ml NaCl 0.45%. Creatinine and cystatin C serum levels were collected one hour before CT-Scan and at day 2, 4 and 10. The T0 value for creatinine and cystatin C was the value measured before the CT-Scan. Serum Creatinine and Cystatin C were measured by the Jaffe method, and by a nephelometric assay respectively.

### Outcome measures

The primary endpoint of the study was the occurrence of contrast nephropathy at day 2, 4 or 10, which was defined as an increase of at least 25% or 44 μmol/l in serum creatinine level or 25% increase in cystatin C levels at day 2, 4 or 10 compared to day 0. We also assessed the proportion of patients with contrast nephropathy regarding the biomarkers used and according to AKIN criteria. The AKIN criteria defined a stage 1 AKI as an increase in creatinine between 150% and 199% from baseline or an absolute increase of at least 26.2 μmol/l, a stage 2 AKI as an increase between 200% and 299% from baseline and a stage 3 AKI as an increase of at least 300% from baseline or a creatinine concentration higher than 354 umol/l with an acute rise of at least 44 μmol/l or initiation of RRT [22].

Secondary endpoints were the mean increases in creatinine and cystatin C concentrations on days 2, 4 and 10 along with the maximum increase during the time periods from day 2 to day 10 (peak increase).

### Statistical analysis

Based on a previous study [17], the proportion of patient with nephropathy was estimated to be 15% in NAC group and 33% in control group. To achieve a power of 90% and a two-tailed risk alpha, the minimal required sample size has been estimated to 106 patients (Fisher’s exact test).

All analyses were performed on an intention-to-treat basis. Data were described as mean +/− SD. Proportions of patients in the control and the NAC groups were compared using Fisher’s exact test. Mean increases in creatinine and cystatin C concentrations were compared between the two groups by use of Mann–Whitney test. Sensitivity analyses were performed to assess the possible effects of loss to follow-up or death on the primary analysis. Pearson’s correlation coefficient was computed between creatinine and cystatin C measures for each day. The significance level was fixed to 0.05 (two-tailed). All statistical calculations were performed using R for Windows (Version 2.11.1, R Development Core Team, 2010).

## Results

### Data collection

**Figure 1:**
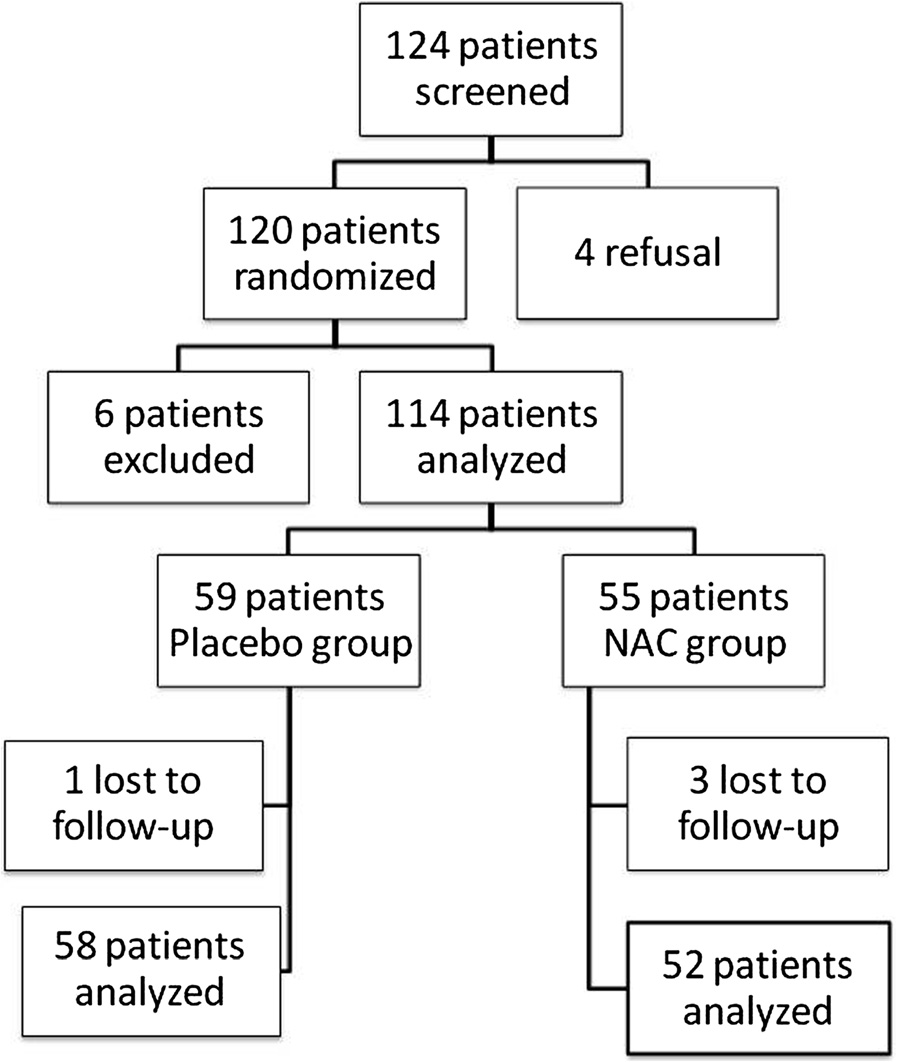
Flow-chart summarizing the study population.

Of the 120 randomized patients (Figure 1), 6 were excluded of the study because they did not undergo contrast CT (n = 4) or presented with a renal obstruction (n = 2).

Follow up did not allow qualification of contrast nephropathy in 4 patients (one in the placebo group, three in NAC group) because they died at day 0 (n = 2) or where lost immediately to follow up (n = 2). These patients were excluded from the main analysis but included in the sensitivity analysis.

In the remaining 110 patients, 8 patients did not have creatinine and cystatin C measured at day 4 either because they died before (n = 4), or because follow up was interrupted (n = 2) and two for technical reasons. Cystatin C at day 4 was not obtained in 7 other patients.

Eleven patients did not have creatinine and cystatin C measurement at day 10, either because they died before (n = 6), or they could not be reached (n = 4) or for technical reasons (n = 1). Seven other patients did not have cystatin C measured at day 10.

### Baseline patient characteristics

At randomization, patients’ demographic and clinical characteristics were similar between the two treatment groups. Treatment medications were also similar between the groups. All baseline parameters are shown in Table 1. Mean estimated GFR according to MDRD was 42 ml/min/1.73 m^2^. Sixty-nine (61%) of the 114 patients had a eGFR < 45 ml/min/1.73 m^2^; 10 (9%) had eGFR < 30 ml/min/1.73 m^2^ on admission.

**Table 1:** Patients characteristic with regard to the inclusion groups

**Characteristics**	**Placebo (n** = **59)**	**NAC (n** = **55)**
Age (years)	78.2 ±11.8	78.1 ± 12.0
Sex (M/F)	29/30	28/27
BMI (kg/m2)	24.4 ± 4.6	25.0 ± 4.8
Diabetes	11 (19%)	15 (27%)
Blood pressure (mmHg)		
Diastolic	69.1 ± 11.7	67.0 ± 17.2
Systolic	129.5 ± 20.8	132.9 ± 22.5
Contrast medium (mL)	117.7 ± 3.2	117.4 ± 1.8
Creatinine (μmol/L)	133.5 ± 34.8	132.4 ± 34.8
Cystatin C (mg/L)	1.9 ± 0.5	1.9 ± 0.6
eGFR (MDRD) (ml/min/1.73)	41.7 ± 1.2	42.7 ± 1.2
Antibiotics	16 (27%)	16 (29%)
Angiotensin-converting enzyme inhibitor (ACE-1 inhibitor)	28 (48%)	25 (45%)
Non-steroidal antiinflammatory drugs (NSAIDS)	1 (2%)	5 (9%)
Diuretics	27 (46%)	25 (45%)

### Outcome

Of the 114 randomized patients, data were available for 110 patients. Out of these 110 patients, 52 received NAC and 58 the placebo. The number of patients who developed contrast nephropathy ranged between 16% (18/110) and 24.5% (27/110), depending on the definition of contrast nephropathy (Table 2). There was no difference between the placebo and the NAC groups, irrespective of the definition of contrast nephropathy.

**Table 2:** Occurrence of contrast nephropathy (several definitions) according to the group of inclusion (placebo or n-acetylcysteine)

**Event**	**Placebo**	**NAC**	**P-value**
	**(n** = **58)**	**(n** = **52)**	
25% increase of creatinine or cystatin C	13 (22.4%)	14 (26.9%)	0.66
25% increase creatinine	10 (17.2%)	8 (15.4%)	0.99
25% increase cystatin C	9 (15.5%)	9 (17.3%)	0.99
AKIN stage 1	11 (19.0%)	13 (25.0%)	0.49
AKIN all stages	12 (20.7%)	13 (25.0%)	0.65

**Figure 2:**
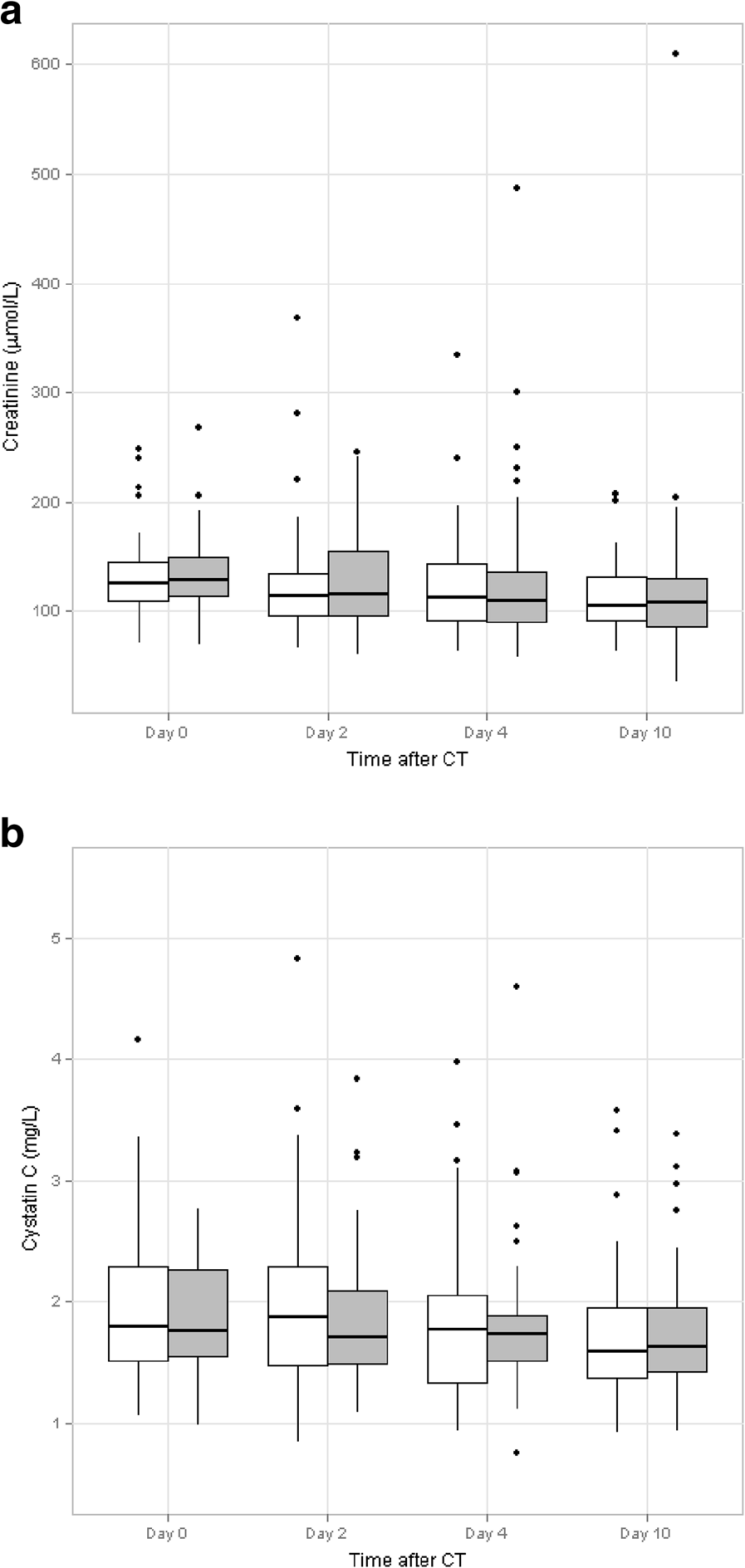
**Median and dispersion of the evolution of creatinine and cystatin C after CT.** (**a**) Median and dispersion of the evolution of creatinine at day 0, day 2, day 4 and day 10 after CT in the N-acetylcysteine group (white) and in the control group (grey); (**b**) Median and dispersion of the evolution of cystatin C at day 0, day 2, day 4 and day 10 after CT in the N-acetylcysteine group (white) and in the control group (grey).

The variations in creatinine and cystatin C concentrations on days 2, 4 and 10 in both groups are reported in Figure 2. The evolution from baseline to day 10 in creatinine and cystatin C levels concentration was not different between the two groups. At Day 10, both cystatin C and creatinine were lower than baseline suggesting that acute renal insufficiency was participating in the renal impairment observed at day 0 (Table 3).

**Table 3:** Mean increases in creatinine and cystatin C on days 2, 4 and 10 compared with day 0, in the two groups

	**Placebo**	**NAC**	**P-value**
	**(n** = **58)**	**(n** = **52)**	
**Day 2**			
Creatinine	−3.60 ± 35.84	−3.66 ± 38.69	0.96
Cystatin C	−0.02 ±0.43	0.06 ± 0.44	0.30
**Day 4**			
Creatinine	−8.25 ± 65.62	−8.37 ± 36.52	0.37
Cystatin C	−0.04 ± 0.57	−0.04 ± 0.41	0.30
**Day 10**			
Creatinine	−14.94 ± 75.53	−16.53 ± 25.23	0.25
Cystatin C	−0.12 ± 0.54	−0.07 ± 0.34	0.27
**Peak increase**			
Creatinine	9.38 ± 70.91	4.63 ± 38.14	0.54
Cystatin C	0.06 ± 0.56	0.17 ± 0.43	0.07

None of the sensitivity analyses, performed to explore the impact of loss to follow-up or death, led to a quantitatively different result: all p-value ranged between 0.30 and 0.99. In particular, the composite event of death or acute kidney injury occurred in 14 (24%) of 58 patients (after exclusion of the patient lost to follow-up) in the placebo group, and in 18 (33%) of 54 patients in NAC group (including the 2 patients who died at day 0).

A correlation coefficient was computed between creatinine levels and cystatin C levels at day 2, 4 and 10. The correlation was of 0.87 at day 2, 0.83 at day 4 and 0.67 at day 10 demonstrating a good congruence of the two measures.

No side effects attributed to NAC injection were observed throughout the study.

## Discussion

This study demonstrates that ultra-high dose intravenous NAC does not prevent contrast-induced nephropathy in patients with impaired renal function undergoing emergency contrast CT-Scan, whatever the definition used for the condition and independently of the use of creatinine or cystatin C as markers of renal function.

In this work, we used both the classical definition of contrast nephropathy (25% increase in serum creatinine or absolute increase of 44 umo/l of creatinine), as well as cystatin C and AKIN criteria. AKIN critera have been recently demonstrated to be predictive of hospital mortality contrast nephropathy in ICU patients [7]. The use of different definition did not modify the fact that iv NAC did not protect against contrast nephropathy, but altered the prevalence of events in our population as it could range from 16 to 24.5% depending on the definition used. More studies are needed to determine which definition best predicts mortality in ER patients after contrast injection.

This study focuses on a poorly studied and fragile population, namely patients from the ER with elevated creatinine levels undergoing emergency CT-Scan. This population is important since it is a large population in which standard preparation before contrast injection is impossible due to time constraints. In addition these patients usually require rapid and often multiple radiologic examinations. Furthermore, appreciation of the basal renal function of these patients is difficult given a usually unique measurement of creatinine level available. In this study, we observe that approximately one out of four patients presenting to the ER with an elevated creatinine undergoing contrast CT-Scan level will develop contrast nephropathy. This high incidence differs from the incidence described post angiography or post elective CT [23], demonstrating that emergency CT in a high risk population is still an important cause of AKI. It is however probable that a significant percentage of these observed AKI are multifactorial. Indeed, according to the severity of the medical conditions and the emergency setting, associated ischemic AKI might contribute to this high incidence. The study was not designed for this purpose but each patient was treated according to the medical illness necessitating the emergency CT SCAN, which includes treatments to prevent or minimize AKI. This is illustrated by the decrease in mean creatinine at Day 10 as compared to baseline creatinine. Nevertheless, we do not think that it does change the conclusion of the study regarding the role of NAC in this setting as our results demonstrate than NAC administration does not alter this renal evolution. The search for efficient and rapid means of prevention of contrast nephropathy in patients with decreased renal function undergoing emergency CT is therefore of paramount importance and should take into account the risk of multifactorial AKI.

NAC possesses potential anti-oxidant and hemodynamic properties that have been hypothesized to protect against contrast nephropathy. Since 2000 and the princeps study from Tepel [13], it has been used extensively for renal protection together with hydration before contrast medium use. However, data in the literature are variable and even meta-analysis are conflicting due to variation of protocols used, heterogeneicity of studies and to publication bias toward positive studies in either oral or intravenous NAC administration [15,24-27]. Moreover the largest trial on oral NAC to date observed no effect of this drug in preventing contrast nephropathy post angiography [28]. However Marenzi and collegues describe a dose dependent protection of NAC, potentially contributing to the variability of results [17]. High doses (6000 mg over 48 h, the first 1200 mg iv before contrast media, 4800 mg orally after contrast media) seems indeed to give a better protection than low doses (3000 mg over 48 h). This may be related to a lower antioxidant effect due to a lower first pass liver generation than in the oral route. This study is the first one focusing on ultra high dose intravenous NAC in the ER population in prevention of contrast nephropathy. Our observation does not substantiate a beneficial role of even ultra high doses of intravenous NAC, administrated within 1 hour before CT, for preventing CN as this treatment was unable to decrease the rate of contrast-induced nephropathy after emergency CT. Although this therapy may appear interesting given its low price and few secondary effects, its administration may result in a false security for the team in charge. This may also lead to under consideration of alternative imagery method or delaying contrast injection in this population. Proper hydration stays the main therapy also in the emergency setting. A 0.9% saline infusion is generally recommended for the hydration of patients with renal failure prior and after injection of contrast media [29]. However, in an emergency setting, when large quantity of fluids have to be administrated in a short period of time, in patients with uncertain cardiac function, we consider that the use of a 0.45% solution [30] is more appropriated to avoid acute pulmonary edema.

A role of NAC on creatinine secretion independently of kidney filtration has been reported by Hoffman and collegues [31]. To correct for this, we also measured cystatin C at all time points of our study. We observed no effect of NAC on AKI based on creatinine or cystatin C level. Furthermore, cystatin C and creatinine levels showed a good correlation at all time points, arguing against a major role of intravenous NAC on creatinine secretion.

Our study is limited by some aspects. First our population is relatively small and we cannot exclude a minor effect of NAC that might have been detectable in a larger cohort. However, such a small effect would probably not be relevant in clinical practice. Second our selection criteria were based on a single creatinine level measurement in the ER. We may therefore have mixed acute and chronic cases of renal impairment, rendering our population less homogenous than in previous studies but closer to everyday practice in the ER. The high rate of CN demonstrates that this population was in any cases vulnerable. Finally, the contrast medium dose used for CT-Scan is generally lower than for cardiac angiography which has been the situation mostly studied regarding NAC effect. Despite these limitations, the follow up of patients with two renal markers, the very high dose of NAC and different definitions of AKI used are strong arguments against an effect of IV NAC on AKI prevention in patients presenting on the ER with a decreased estimated clearance and undergoing contrast CT-Scan.

## Conclusions

In summary, contrast-induced nephropathy incidence is high in patients with a low estimated GFR undergoing an emergency CT-Scan. Intravenous NAC at high dose is not more efficient in this population than hydration alone for prevention of contrast nephropathy and should not be used in this setting.

## Competing interests

The authors have no commercial, financial or any other interest to disclose with regard to the current submission.

## Authors’ contributions

PAP, AP, and PYM did conceive the study. PAP, AP, SDS, FS, CDB, PS and PYM did equally contribute to the design of the study. PAP and AP did collect the data and obtained the informed consent. EDL and TP performed the statistical analysis of the data. PAP, AP, EDL, TP and PS carried the data analysis and interpretation. PAP, AP, SDS, FS, CDB and PYM did participate to the drafting of the manuscript. PAP, CDB and PYM did participate to the coordination of the whole study. All authors read and approve the final manuscript.

## Authors’ information

PAP and AP are specialized in emergency radiology. They are members of a research team in this field. They are particularly concerned by the optimization of the protocols in the emergency department, to improve the quality of care and efficiency in managing emergency patients. SDS, PYM and PS are specialized in clinical nephrology. They are in charge of the treatment and the prevention of acute renal failure in patients admitted in our hospital. Thus, they are particularly concern by contrast nephrotoxicity in vulnerable patients. CDB is head of the radiology department. EDL and TP are specialists in epidemiology and statistics.

## Pre-publication history

The pre-publication history for this paper can be accessed here:

http://www.biomedcentral.com/1471-2369/14/119/prepub
